# Exploring the Potential of T-Cells for a Universal Influenza Vaccine

**DOI:** 10.3390/vaccines8040598

**Published:** 2020-10-11

**Authors:** Sharon Vijayanand, Keegan Braz Gomes, Rikhav P. Gala, Mohammad N. Uddin, Martin J. D’Souza

**Affiliations:** 1 Vaccine Nanotechnology Laboratory, Mercer University, Atlanta, GA 30341, USA; sharon.c.p.vijayanand@live.mercer.edu (S.V.); Keegan.Brazgomes@live.mercer.edu (K.B.G.); uddin_mn@mercer.edu (M.N.U.); 2 Fraunhofer USA, Center for Molecular Biotechnology, Newark, DE 19711, USA; rikhav.praful.gala@gmail.com

Among the four types of influenza viruses, the influenza A strains and their subtypes have been responsible for causing worldwide pandemics and seasonal epidemics. Around 131 subtypes of the Influenza A virus have been detected and have had their viral genomes mapped [1]. However, creating a vaccine for each specific subtype of the virus is impractical. Moreover, the efficacy of currently existing subtype-specific vaccines, although satisfactory, is affected by several undesirable factors such antigenic drift, mismatch, and pandemic emergence due to assortment. These vaccines are unable to protect most of the population in the event of an emerging novel pandemic strain. Therefore, developing a universal vaccine which would provide cross-protection against the most prominent influenza strains and their subtypes is critical in the near future. 

When vaccinated against a specific disease, the adaptive immune system in the body is activated, which is comprised of the T cell and B cell-mediated responses. Therefore, when the actual pathogen attacks the body, it is recognized by the immune system, resulting in a stronger, faster and antigen-specific response, eliminating the invading pathogen. This is the basis of adaptive immunity and T cells play a key role in this process. Recent research has provided promising evidence that T cells evoke a protective as well as long-term immune response [2], capable of mediating heterosubtypic immunity by the recognition of antigenic epitopes conserved across different viral strains and subtypes of the inactivated A virus (IAV) [3]. T cells include the cytotoxic CD8^+^ T cells and the helper CD4^+^ T cells, which recognize the antigenic peptides present on the surface of the antigen-presenting cells (APCs) via the major histocompatibility complex (MHC) molecules [4]. In case of a pandemic or seasonal antigenic drift, there may be an insufficient antibody response capable of fighting the infection. In contrast, a T cell vaccine has the potential to produce a memory T cell response, which can recognize a range of dissimilar and even unrelated influenza viruses, by targeting linear peptide sequences conserved across varying influenza virus strains and subtypes [5,6,7,8,9,10]. A preclinical animal study reported that no detectable virus replication was observed when mice were primed with the H3N2 virus, boosted with the H1N1 virus and challenged with the H7N7 virus, demonstrating heterosubtypic protection provided by memory T cells [11]. Accordingly, designing a vaccine that will induce a prominent T cell response, which in turn produces heterosubtypic immunity in an individual, is the basic theory underlying the idea of ‘a universal vaccine’ [3,5].

Several fundamental factors should be taken into consideration prior to developing a universal vaccine, which harnesses the power of T cells. Current vaccine strategies involve the use of surface proteins for vaccine development, which inhibits viral infection in the host but fails to produce conserved T cell epitopes. In contrast, internal proteins are more effectively conserved between different IAV strains and their subtypes, which is of much interest in cross-protection [12,13]. The existing data from human studies report the limited efficacy of both the inactivated influenza vaccine (IIV) and live attenuated influenza vaccine (LAIV), which should be considered while developing a T cell-inducing vaccine [14]. 

CD4^+^ helper T cells have also been shown to be critical for establishing both T cell and B cell immune memory. Furthermore, studies in mouse models have shown that CD4^+^ T cells also provide protection from heterologous influenza infection, which makes CD4^+^ T cells imperative to future vaccine design [12]. Additionally, the imprinting of hemagglutinin (HA)-subtype-specific CD4^+^ and B memory cells after prior exposure to various influenza strains can result in the protection from severe infection from similar strains in the future [15]. CD4^+^ T cells have been shown to offer protection against infection with heterologous influenza strains through their direct cytotoxicity towards influenza infection [16] and reduction in viral loads [17]. Therefore, the effector functions of CD4^+^ cells including the recalling of heterologous T and B cell responses is highly desirable for developing a universal influenza vaccine.

The role of the T cells in reducing the severity of flu symptoms is supported by ample clinical data. However, standard assay methods to identify a T cell trait that can be attributed to protection is still lacking. T cells act locally, generating a T cell-mediated response when they encounter target cells carrying the antigenic peptide. As the point of entry of the influenza virus is via the respiratory tract, a previously deposited pool of resident memory T cells in the mucosal tissue of the respiratory tract will aid in the prevention of infection and facilitate the elimination of the virus. Tissue resident memory (Trm) in the respiratory tract and lungs respond rapidly to infection by inducing high levels of cytotoxic mediators and effector molecules targeting the virus [18]. An effective vaccine that induces strong influenza virus-specific Trm is a key consideration for developing an effective T cell vaccine. As mentioned previously, internal viral proteins such as the nucleoprotein (NP), polymerase basic 1 protein (PB1), and matrix protein 1 (M1) are highly conserved across different strains of the influenza virus and are therefore capable of producing cross-strain immunity, which is necessary for developing a universal vaccine [5]. Although humans exhibit a diverse expression of MHC alleles and a human leukocyte antigen system (HLA) that varies across ethnicities, T cells generally focus on a select number of peptide + HLA epitopes. However, the search for a universal vaccine should address the current existing ethnic bias in HLA profiles of diverse populations. Variations in T cell responses can be observed depending on the type and class of HLA alleles expressed by the individual [7]. Therefore, studying the HLA profiles of ethnically diverse individuals can contribute to the development of a T cell vaccine that can produce a cross-reactive T cell response, activating multiple immune mechanisms and resulting in an overall robust heterologous immunity.

The influenza virus also contains mechanisms that allow viral escape from the immune-mediated control, which limits the potential of a truly universal influenza vaccine. For example, CD8^+^ T cell escape has been seen in certain T cell epitopes of naturally circulating influenza virus subtypes [19]. Additionally, preliminary results have confirmed that influenza viral escape can occur very quickly after the virus has infected a mouse [20]. However, evidence suggests that HLA frequencies in a cell population contribute to the rate of T cell-mediated immune escape. To circumvent this issue, various T cell sets can be primed against variants that are likely linked to immune escape [21]. The resulting vaccine strategy would incorporate a modified mix of peptides that could reduce the likelihood of viral escape. However, the specificity of HLA selection for determining the full-length proteins may be unrealistic for large-scale vaccine production. A recent study demonstrated that children infected with human immunodeficiency virus (HIV) were able to generate stronger HIV variant-specific CD8^+^ T cell responses compared to HIV-infected adults. A similar trend has been reported in children aged 4–14 that have been infected with naturally circulating influenza. Hence, researchers are exploring the significance of combining T cell epitopes, TCR escape variants, and the time of vaccination to produce stronger T cell-inducing influenza vaccines. However, current clinical development does not emphasize the potential of T cell immunity and should therefore be further explored. Certain strategies in clinical development include the use of vectors such as Modified Vaccinia Ankara (MVA) and Simian Adenovirus-encoding internal NP and M1 influenza proteins, which have shown potential to produce robust immunity. The MVA+NP/M1 vaccine has shown positive results in adult and elderly populations and is protective against influenza [22]. T cell-based vaccine strategies have also been evaluated in ferret models, which have shown reduced transmission and lower peak viral loads in vaccinated ferrets [23]. Additionally, viral vectors may possess a self-adjuvanting quality, reducing the need of exogenous adjuvants. Recently, squalene-oil-in-water-based adjuvant MF59 has been approved for use in marketed influenza vaccines and has been reported to improve CD4^+^ T cell responses post-vaccination [24,25,26]. The use of adjuvants demonstrated strong CD8^+^ cell responses in ferrets, however, it has not demonstrated the same efficacy in human populations [27].

The review highlighted the underutilized potential of influenza-specific T cell immunity in designing a potential universal vaccine. However, there are some limitations of a T cell approach such as the short life of resident T cells, viral escape, and roles of HLA alleles in population coverage. Also, assessing T cell memory that is influenza-specific in human subjects is more demanding compared to the serology approaches used for assessing antibody responses such as hemagglutination inhibition (HAI) assays. Nonetheless, the potential for T cell recognition against influenza viruses is immense. Antigen development research must also be explored in greater detail to understand its true potential for its application in the development and screening of true universal influenza vaccine candidates that are efficacious and cross-protective against a broad range of influenza strains and their subtypes.
